# Pilot Study in Investigating Material Financial Toxicity Markers by Age in Cancer Patients

**DOI:** 10.1089/jayao.2022.0011

**Published:** 2023-02-14

**Authors:** Amber Skinner, Melanie Buhlmann, Brooke L. Fridley, Damon R. Reed, Deborah Vicedo, Neil T. Mason

**Affiliations:** ^1^ Department of Individualized Cancer Management, H. Lee Moffitt Cancer Center and Research Institute, Tampa, Florida, USA.; ^2^ Department of Adolescent and Young Adult Program, H. Lee Moffitt Cancer Center and Research Institute, Tampa, Florida, USA.; ^3^ Department of Biostatistics and Bioinformatics, H. Lee Moffitt Cancer Center and Research Institute, Tampa, Florida, USA.; ^4^ Cancer Biology and Evolution, H. Lee Moffitt Cancer Center and Research Institute, Tampa, Florida, USA.; ^5^ Patient Financial Services, H. Lee Moffitt Cancer Center and Research Institute, Tampa, Florida, USA.; ^6^ Precision Medicine Program, H. Lee Moffitt Cancer Center and Research Institute, Tampa, Florida, USA.

**Keywords:** adolescent and young adult, Affordable Care Act, financial toxicity, sarcoma

## Abstract

**Purpose:**

Studies have shown that financial toxicity can reduce survival, decrease quality of life, and reduce compliance with treatments. The aim of this retrospective study was to investigate material markers of financial toxicity, including insurance coverage, financial assistance, and balances due among adolescent and young adult (AYA) (18–39), adult (40–64), and senior adult (>65) patients with a sarcoma diagnosis after the Affordable Care Act became effective.

**Methods:**

This study performed a retrospective analysis of possible indicators within the material domain of financial toxicity in sarcoma patients, a common diagnosis in young adult patients. Indicators of financial toxicity included: insurance status and number of insurances, charity care, accessing financing options, or having an unpaid balance referred to a collection's agency.

**Results:**

The cumulative charges per patient were similar between AYA, adult, and senior adult populations at an average of $194,329 (standard deviation [SD] = $321,425), $236,724 (SD = $368,345), and $188,030 (SD = $271,191), respectively. AYA patients were more likely than adult and senior adult patients to have Medicaid coverage (income-based government insurance) (22.1% vs. 8.4% vs. 1.2%), receive charity care (5.3% vs. 2.6% vs. 1.2%), or have a balance referred to a collection's agency (9.2% vs. 5.8% vs. 1.2%).

**Conclusions:**

This study suggests that younger cancer patients are more likely to suffer material financial strain and additional financial resources may need to be made available to ensure they can receive care without an increase of financial toxicity markers and undue financial stress.

## Introduction

There are over 89,000 new cancer diagnoses in adolescents and young adults (adolescent and young adult [AYA]; 15–39 years of age) in the United States each year according to the National Cancer Institute (NCI). The development stage for AYAs include both biological and social role development transition and contributes to the unique needs of the AYA cancer population.^1^ Although AYA survival rates within different cancer diagnoses continue to lag behind younger and older patient cohorts, recognition of AYA oncology as a distinct unique cohort has improved outcomes for this population.^2^ Research has suggested an age-appropriate, multidisciplinary approach to addressing the pillars of AYA oncology from diagnosis through survivorship due to the multifactorial challenges AYA cancer patients and survivors encounter, such as, compliance issues, financial hardship, and low accrual rates to clinical trials.^3–5^ Financial challenges among the AYA population is an essential pillar to explore as cancer treatment and associated costs are expected to rise increasing the risk of financial hardship and toxicity for AYA cancer patients and survivors.^6,7^

Financial toxicity broadly refers to the financial consequences of high out-of-pocket health care expenses, in which studies have shown cancer patients to obtain, and subjectively to the psychosocial impact of those financial consequences.^7,8^ The NCI defines financial toxicity as the cost of treatment that leads to financial issues for patients.^9^ Higher medical costs that induce a burden on patients can affect compliance, health-related quality of life, lead to debt or bankruptcy, and negatively impact clinical outcomes.^8,10–13^ Research has illustrated multiple domains of financial hardship for cancer survivors, including material (out-of-pocket expenses, loss of income), psychological (financial distress), and behavioral (not adhering to treatment plan due to financial hardship) aspects.^14^ Due to the development stage of transitioning into independence and the lack of financial security compared with older adult cancer patients or pediatric patients with parental support, AYA patients have been shown to have higher material financial toxicity indicators.^15,16^

Young adult cancer patients represent the highest percentage of the uninsured cancer population, particularly those ≥26 years of age, which can lead to high out-of-pocket costs, cost-associated unmet care needs, and inconsistent follow-up care.^15^ Affordability of insurance coverage within the AYA age group remains a challenge, even after the Patient Protection and Affordable Care Act (ACA) became effective in 2010, with the expectation to increase access to affordable health care and insurance for the uninsured U.S. population.^17^ Young adult cancer patients are also more likely to be unemployed than older adult (>40) cancer patients.^18^ Cancer during adolescence and young adulthood can have a significant influence on the survivor's ability to work, career development, and potential earnings, in which the duration of this long-term effect is extended for AYAs compared with older patients.^3,19–21^ An additional AYA specific cost is fertility preservation before treatment, which is often an out-of-pocket cost and only specific to cancer patients of child-bearing age.^19^

Others have reported on the financial hardship and evaluated cancer-related medical debt and the overall mental effects of the financial burden of either all cancer survivors or survivors with the most common cancer diagnosis (i.e., lung, breast, or colon cancer), but to our knowledge, there has not been a focus on rarer cancers like sarcoma that impact patients of all ages, including AYAs.^22–25^ Sarcomas are often treated with multiple modalities, including surgery, chemotherapy (often inpatient) and radiation, which would be predicted to enrich for higher total costs of care.^26^ Additionally, sarcomas are a rather heterogeneous group of diagnoses with prognosis heavily influenced by presenting stage with metastatic presentations difficult to cure and localized patients with a typical long-term survival of about 70%.^26,27^

In addition, the majority of studies have focused on either total medical expenditures of cancer patients compared with those without cancer based on Medical Expenditure Panel Survey data or patient-reported data, in which treatments or living expenses are forgone or negative financial impacts like bankruptcy.^7,8,22–25,28^ These studies capture extremely important indicators of financial toxicity; however, they are unable to quantify the amount of medical expense that leads to negative financial outcomes or additional markers of financial need like payment plans and the provision of charity care for those in need.

The aim of this retrospective study was to investigate markers of material financial toxicity, after ACA became effective, utilizing data available within a comprehensive cancer center, including insurance type and coverage over time, financial assistance, credit-worthy financing options and balances due, between AYAs (18–39), adults (40–64), and senior adults (>65) with a sarcoma diagnosis between 2012 and 2018. We hypothesized that AYA sarcoma patients and survivors will have more indicators of material financial toxicity than adults and senior adults, despite the amount of medical charges compared and ACA provisions.

## Methods

A retrospective review was performed of all sarcoma patients diagnosed between January 1, 2012 and December 31, 2018. The study was performed at an NCI-designated cancer center in Tampa, Fl with a catchment area of 15 counties and offering services to 6,145,524 residents, 29% of Florida's population.^29–31^ Demographic and clinical data were collected from the Center's Cancer Registry, that is, age at diagnosis, sex, race, tumor stage, and vital status. The Cancer Registry is a comprehensive data set on over 211,000 eligible patients diagnosed and/or treated at the cancer center.^29^ The data are reported to the Florida State Cancer Registry (FCDS) and the National Cancer Database (NCDB), which is developed and maintained by the American College of Surgeons. The Registry includes demographic data, medical history, diagnostic findings, cancer information, and cancer treatment. ICD diagnosis codes ICD9-176 and ICD10-C46 were used to identify patients in the cancer registry who qualify for inclusion in the study.^14^ Eleven patients did not meet reportability criteria for inclusion in the registry and were excluded from the study.

Financial and insurance coverage data, that is, total charges, insurance status inclusive of all changes over the interval of data collected, utilization of third-party financing, and referral to collection agencies were collected from Soarian, a clinical workflow tool designed to streamline the collection of financial data as charges occur and manage clinical information and data. Charge-level billing data by individual patient were obtained beginning at date of diagnosis through April 20, 2020. Medical charges were used to compare the total cost of care for sarcoma between age cohorts. The study was approved by the cancer center Scientific Review Board and determined to be exempt by the Institutional Review Board.

Patients were grouped by age at diagnosis for comparison: AYA, 18–39 years; middle aged adult, 40–64 years; and senior adult, 65+ years. Total charges per patient were calculated from charge-level billing records over time, out-of-pocket costs were not available. Insurance status and changes to insurance (e.g., change of insurer or loss of insurance coverage) were determined from billing records. Insurance coverage was classified by commercial, government (e.g., Medicare and Medicaid), self-pay, or charity.

**FIG. 1.**
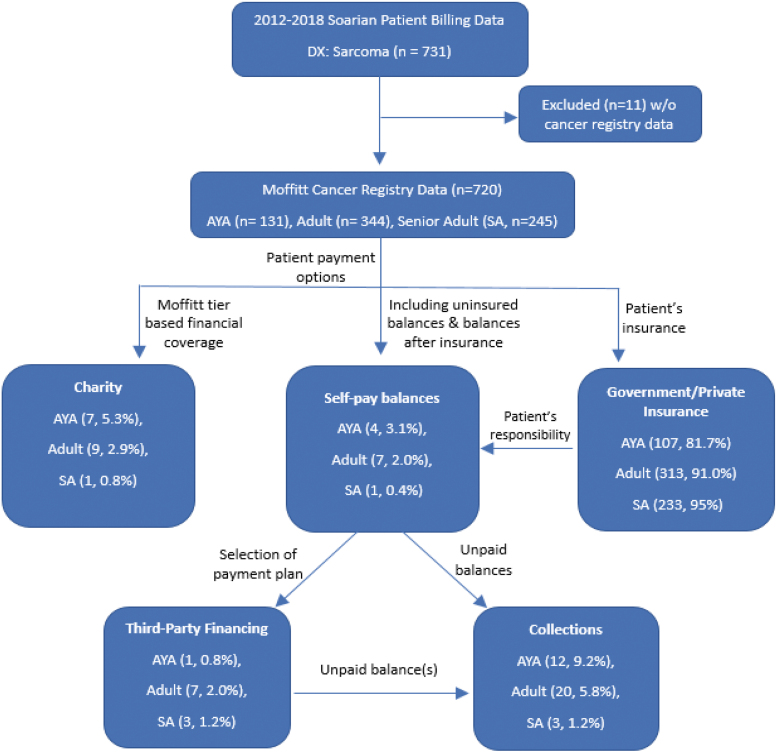
Study participants and insurance type relationship between age cohorts, payment options, and third-party financial services.

Indicators of financial toxicity include balance write-off by institutional charity care, utilization of a third-party financing program offering debt to be paid to cancer care institution (i.e., AccessOne^®^), and/or referral to a third-party debt collection agency (Fig. 1). Charity care is a center-funded allocation to patients who qualify for low-income criteria based on federal poverty guidelines and receive financial assistance for their medical cancer care expenses. Third-party financing was patient selected and only approved to patients with previous favorable credit to provide payment plans for unpaid balances. Past due unpaid balances with or without a selection to third-party financing were sent to collections.

Comparative statistics across age groups were calculated using analysis of variance (ANOVA_ for continuous measurements and Chi-square or Fisher's exact for categorial variables. Pairwise comparisons between two age groups were completed using two-sample *t*-tests or Chi-square/Fisher's exact test. Results were considered statistically significant if *p* < 0.05. All statistical analyses were performed using R (version 4.1.0).^32^

## Results

Seven hundred twenty patients treated for sarcoma at the NCI-designated cancer center with available financial and cancer registry data were identified between January 1, 2012 and December 31, 2018. Patients were categorized by demographics, stage, and insurance status.

No statistically significant difference was observed between age groups based on gender or stage of disease (Table 1). The AYA cohort was demographically similar to older cohorts aside from age and racial diversity. Adult and senior adult populations were similar in racial composition with AYA populations having a significantly higher percentage of non-Hispanic Black AYA patients at 18.3% compared with 9.9% and 7.8%.

**Table 1.** Demographic, Disease, and Financial Characteristics of Patient Population

Characteristic	Overall	AYA	Adult	Senior adult	*p*	*n*
	*n* = 720 (%)	*n* = 131 (%)	*n* = 344 (%)	*n* = 245 (%)		
Sex^a^					0.011	720
Female	368 (51.1%)	60 (45.8%)	196 (57.0%)	112 (45.7%)		
Male	352 (48.0%)	71 (54.2%)	148 (43.0%)	133 (54.3%)		
Race^b^					0.001	720
American Indian	3 (0.4%)	2 (1.5%)	0 (0.0%)	1 (0.4%)		
Asian	24 (3.3%)	8 (6.1%)	11 (3.2%)	5 (2.0%)		
Black	77 (10.7%)	24 (18.3%)	34 (9.9%)	19 (7.8%)		
Other/unknown	32 (4.4%)	9 (6.9%)	16 (4.7%)	7 (2.9%)		
White	584 (81.1%)	88 (67.2%)	283 (82.3%)	213 (86.9%)		
Hispanic origin^b^					0.058	720
Hispanic	79 (11.0%)	20 (15.3%)	43 (12.5%)	16 (6.5%)		
Non-Hispanic	638 (88.6%)	110 (84.0%)	300 (87.2%)	228 (93.1)		
Unknown	3 (0.4%)	1 (0.8%)	1 (0.3%)	1 (0.4%)		
Median age at diagnosis^c^	58.0	29.0	55.0	71.0	<0.001	720
TNM/CS mixed stage^a^					0.421	720
1-2A	186 (25.8%)	35 (26.7%)	89 (25.9%)	62 (25.3%)		
2B-3	186 (25.8%)	36 (27.5%)	87 (25.3%)	63 (25.7%)		
4	141 (19.6%)	21 (16.0%)	73 (21.2%)	47 (19.2%)		
Not applicable	141 (19.6%)	27 (20.6%)	57 (16.6%)	57 (23.3%)		
Unknown	66 (9.2%)	12 (9.2%)	38 (11.0%)	16 (6.5%)		
Vital status^a^					0.001	720
Alive	220 (30.6%)	58 (44.3%)	99 (28.8%)	63 (25.7%)		
Dead	500 (69.4%)	73 (55.7%)	245 (71.2%)	182 (74.3%)		
Insurance type^b^					<0.001	720
Charity	17 (2.4%)	7 (5.3%)	9 (2.6%)	1 (0.4%)		
Commercial	292 (40.6%)	70 (53.4%)	215 (62.5%)	7 (2.9%)		
Medicaid	61 (8.5%)	29 (22.1%)	29 (8.4%)	3 (1.2%)		
Medicare	300 (41.7%)	8 (6.1%)	69 (20.1%)	223 (91.0%)		
Other	38 (5.3%)	13 (9.9%)	15 (4.4%)	10 (4.1%)		
Self-pay	12 (1.7%)	4 (3.1%)	7 (2.0%)	1 (0.4%)		
Third-party financing^b^					0.574	685
Yes	11 (1.6%)	1 (0.8%)	7 (2.2%)	3 (1.2%)		
No	674 (98.4%)	118 (99.2%)	317 (97.8%)	239 (98.8%)		
Collections agency referral^b^					0.002	709
Yes	35 (4.9%)	12 (9.2%)	20 (5.9%)	3 (1.2%)		
No	674 (94.1%)	118 (90.8%)	317 (94.1%)	239 (98.8%)		

^a^ Tested with Chi-square test.

^b^ Tested with Fisher's exact test.

^c^ Tested with Kruskal–Wallis test.

AYA, adolescent and young adult; CS, collaborative staging.

**FIG. 2.**
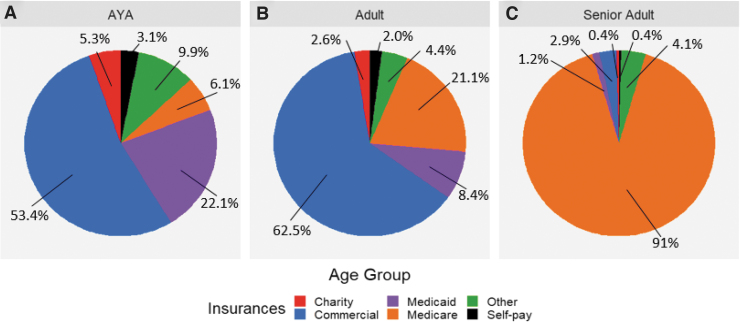
Insurance type by age group **(A)** AYA, **(B)** adult, and **(C)** senior adult. AYA, adolescent and young adult.

**FIG. 3.**
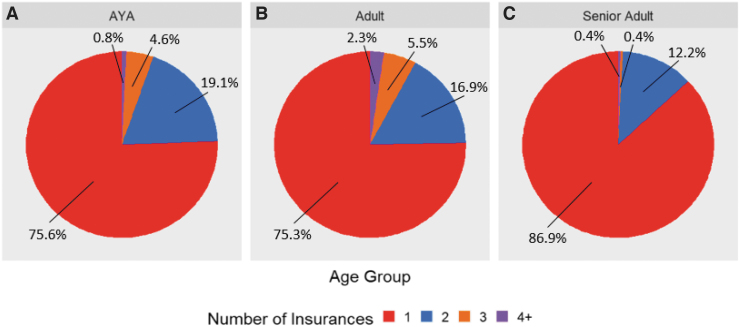
A number of insurances by age group **(A)** AYA, **(B)** adult, and **(C)** senior adult.

A significant difference in insurance providers was observed between age groups (*p* < 0.001). AYA patients were more likely to have Medicaid coverage as well as charity care, self-pay, and other coverage (Fig. 2). The senior adult population had a higher percentage of Medicare coverage and lower percentage of commercial insurance. AYA and adult patients had higher percentages of individuals with multiple insurance policies compared with senior adult patients, the majority of which had only one or two policies (Fig. 3).

**FIG. 4.**
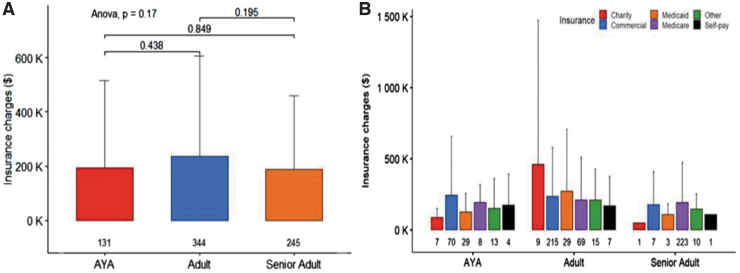
Mean cumulative charges per patient by **(A)** age group and **(B)** age group and insurance type. The study did not include enough patients utilizing financial assistance programs, third-party financing or charity care, to detect statistical differences between age groups.

In looking at the charges per patients, the mean cumulative charges per patient were not significantly different between age groups (Fig. 4 and Table 2) based on pairwise *t*-tests, with charges being a summation of charges over the entirety of care at the cancer center.

**Table 2.** Mean (Standard Deviation) Charges and Balances Due by Age Group

Financial measure	Overall	AYA	Adult	Senior adult
Mean cumulative charges	$212,441 ($330,013)	$194,329 ($321,425)	$236,724 (368,345)	$188,030 ($271,191)
Mean financing balance	$2,325 ($3,894)	$0	$1,366 ($1,996)	$5,337 ($6,657)
Mean collections balance	$1,648 ($1,982)	$1,771 ($2,308)	$1,435 ($1,660)	$2,571 ($3,085)

Data are *n* (SD) unless otherwise stated.

SD, standard deviation.

Although, this analysis is descriptive in nature, trends can be seen suggesting differences between age groups. AYA patients had the highest percentage of charity care and unpaid balances due, which were sent to a collection agency, 5.3% and 9.2% (Table 1). AYA patients were also the least likely to utilize third-party financing (0.8%).

Balance information was a snapshot as of July 23, 2021. There was no remaining third-party financing balance at the time of data analysis for the AYA patient utilizing the third-party financing service while the mean balance for the 12 AYA patients referred to collections was $1,771. The AYA patient cohort had a higher percentage (9.2%) of unpaid balances being sent to collections than the older cohorts. Adult and senior adult patients were more likely to utilize third-party financing (2% and 1.2%, respectively) with mean balances of $1,365 and $5,337, respectively. Seniors also had the highest mean balance referred to collections at $2,571 versus $1,435 for adult patients and $1,771 for AYA patients.

## Discussion

The goal of this study was to investigate material markers of financial toxicity in sarcoma patients across age cohorts after ACA became effective. We found higher rates of financial toxicity markers in the AYA patients even though groups had comparable charges and severity of disease. The AYA population had a higher percentage of Medicaid coverage, changing of insurance types, charity care, self-pay balances, and unpaid balances sent to collections indicating more financial toxicity markers due to cancer care expenses and negative financial consequences due to the inability to pay medical bills.

Quantifying the dollar amount of balances requiring third-party financing or referral to a collection agency was unique to this study and provides perspective on the relatively small balances owed that may result in financial toxicity. Although the third-party financing company offered more affordable payment plans to patients, only one AYA patient utilized the payment option, which could relate to the following indications, either (1) AYAs had less previous favorable debt payment history or lower credit rates or (2) AYAs were unaware of financing option available to them. A higher percentage of AYA patients had unpaid balances requiring referral to a collection agency and had a lower mean balance owed compared with older cohorts despite the amount of total cancer expenses. This indicates that AYA populations may be more susceptible to financial toxicity during cancer care at relatively small balances owed.

This study expands literature illustrating the long-term effects of cancer by identifying another potential negative financial implication as referral to collections and unpaid balances, which can impact a patient's credit score and affect their ability to obtain a loan, rent an apartment, and so forth in the future.^33–36^ The inability for AYA patients to pay even modest outstanding balances makes intuitive sense as these patients have not established their careers, had the opportunity to accrue savings, and may be at higher risk of losing a job due to their illness.^37^

In addition to young adult cancer patients experiencing financial distress throughout their cancer journey, including an impact of earnings, career development, retirement decisions, etc., patients with sarcoma demonstrate one of the lowest return to work rates.^38–40^ Therefore, AYA cancer patients and survivors remain more likely to be impacted by financial toxicity and in need of short- and long-term financial support and navigation, despite federal ACA expansion of health care coverage and access to care. This analysis was conducted in a state that did not expand Medicaid and results may differ in other areas.

Unpaid balances, with or without insurance, are indicative of financial distress as patients are unable to pay for their care in a timely manner. Patients who do not qualify for charity care, in-house payment plans, and are unable to pay their balance due in sufficient time are referred to the third-party financing company, which sets up a financing plan based on the individual's credit worthiness. Although the number of patients in the analysis is relatively small, the higher balance owed for senior adults may indicate (1) senior adults are more aware of the financial assistance resources available to them or (2) they have more available assets to be used as collateral or higher credit scores in general compared with the one AYA patient who utilized the third-party financing service.^20^ The difficulty of navigating health care systems for AYAs could contribute to the lack of AYAs using financial resources available.

Older AYAs have expressed wanting navigation support throughout the cancer continuum with more concerns about their family's wellbeing and finances.^41^ Addressing this navigation need may increase AYA patients’, survivors’, and caregivers' knowledge to better understand insurance coverage and financial resources (e.g., third-party financing), out-of-pocket costs, navigating the financial aspect of the health care system, decrease the likelihood of unpaid balances, and align external health care policy resources.^42^

Passage of the ACA was expected to have a positive impact on cancer care, including expansion of eligibility for Medicaid and dependent insurance coverage up to age 26.^43–45^ Medicaid is a public-funded health insurance program eligible to low-income individuals or families in the United States.^45^ Florida has a higher rate of uninsured individuals than other states, yet the state has chosen not to accept federal funding to expand ACA-eligible Medicaid provisions.^44^ In this study, a higher percentage of AYA patients had Medicaid coverage confirming AYA patients are more likely to have low incomes to qualify for coverage and are at greater risk of financial toxicity due to cancer care expenses. AYA patients are also more likely to be provided charity care based on financial need, which suggests this cohort would benefit from expanded state Medicaid coverage under ACA.

The senior adult cohort had a higher percentage of Medicare coverage, a universal health insurance program for those over the age of 65 regardless of income, younger people with disabilities, and people with End-Stage Renal Disease; and this cohort was less likely to have financial toxicity markers, such as unpaid balances, charity care, self-pay balances, or multiple insurances.^46^ The 6.1% of AYA patients with Medicare most likely qualified because of a disability and a low-income rate, according to Medicare's eligibility requirements, reinforcing the lack of financial security, reduced income, or the inability to work within the AYA population.^16,46^

Contrary to age, the increased racial diversity in the non-Hispanic, Black AYA population at the comprehensive cancer center is consistent with data presented by the recent study by Sultan et al showing that the likelihood of minority patients being treated at an NCI-designated cancer center is reduced as age increases.^47^ The significant increase in utilization of NCI-designated cancer center services in the minority AYA population is encouraging and provides optimistic data of access to care to NCI-designated cancer care for Black AYA patients.

Limitations of the study include the inability to access balances over time for each age cohort and determining personal versus employer-provided insurance plans, as changes in insurance from employer to personal could reveal a potential layer of toxicity between patients with insurance coverage. Patient portion owed of the total cost of cancer care was unavailable due to data sensitivity and practical ability to obtain the data in a reasonable amount of time. Another limitation to the study is the bias of the third-party financing vendor, which became available to patients in October 2018; therefore, patients with self-pay balances before this time did not have access to this financial option and the comprehensive cancer center did not have access to any external financing data.

Additional studies need to be performed to expand on the data presented. Future studies will address the limitations of this study. First, additional diagnoses will be included to increase the population size and provide additional statistical power for analysis. Second, additional material financial toxicity markers, including balance over time, mean time of initiation of payment plan options and to pay off balance, and referral to financing and/or collection agencies are expected to be available. These data points will provide a clearer picture of financial toxicity over time compared with this study that examined balances at a single point in time. Additionally, increasing demographic variables will allow supplemental comparison findings for race and ethnicity, education, martial, and socioeconomic status.

Finally, these results need to be replicated in a larger study of patients with cancer, possibly using a multi-institutional data, including non-NCI-designated cancer centers and private practices to increase the statistical power for relatively infrequent events (i.e., submission of payment to a collection agency) and address the generalizability of the results to the general AYA patient population. The goal of these future studies is to better characterize differences in financial burden to younger patients and inform solutions for additional resources to reduce unnecessary financial strain unique to this population.
